# Continuous Increase in the Framework Flexibility of ZIF-90 with Crystal-Size Reduction to the Nanoscale: Evidence for Missing Metal Nodes Using Single-Crystal Infrared Spectroscopy

**DOI:** 10.1021/acs.langmuir.6c03586

**Published:** 2026-09-10

**Authors:** Akalanka B. Ekanayake, Collin S. Hill, Thumini R. Dias, Leonard R. MacGillivray, Alexei V. Tivanski

**Affiliations:** † Department Chemistry, 4083 University of Iowa, Iowa City, Iowa 52242-1294, United States; ‡ Département Chimie, Université de Sherbrooke, Sherbrooke, Quebec J1K 2R1, Canada

## Abstract

Here we investigate the effects of crystal size on the mechanical properties (Young’s modulus, framework flexibility) of a switchable metal–organic framework (MOF): zeolitic imidazolate framework-90 (ZIF-90) individual crystals with sizes ranging from micro- to nanodimensions. Atomic force microscopy (AFM) nanoindentation measurements over individual crystals reveal a significant reduction in Young’s modulus with crystal-size reduction that follows a continuous power-law relationship, where Young’s modulus decreases by nearly 1 order of magnitude as crystal base size decreases from ∼5 to 0.4 μm. Scanning electron microscopy (SEM) and energy-dispersive X-ray spectroscopy (EDX) analyses coupled with the AFM results reveal that the origin of the size-dependent framework flexibility is primarily attributed to an increased concentration of missing metal node defects for smaller crystals. The presence of missing metal node defects in ZIF-90 crystals was further confirmed by single-crystal optical photothermal infrared spectroscopy measurements. Significantly, the value of the power-law exponent in Young’s modulus versus crystal-size data was related to the magnitude of the relative increase in the number of missing metal node defects, indicating that the extent of the framework flexibility change with crystal size can in part be attributed to the rate of defect accumulation with crystal downsizing at the nanoscale. These findings indicate that the power-law scaling of mechanical properties may serve as an approach to assess the size-dependent extent of point-defect evolution in switchable MOFs, potentially enabling a rational design of solids with controlled functional properties.

## Graphical abstract

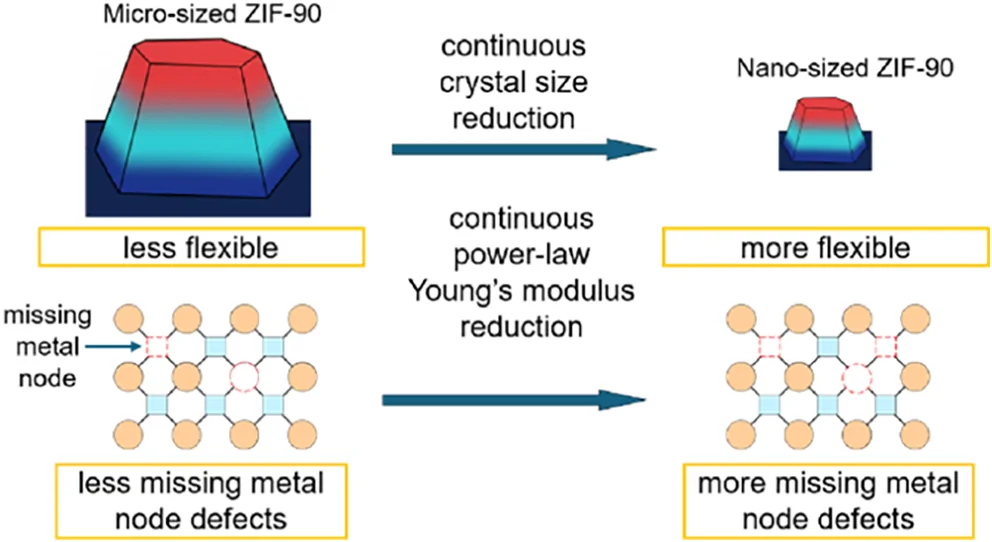

## Introduction

Metal–organic frameworks (MOFs) are crystalline materials composed of metal ions coordinated to organic ligands, forming extended porous networks.^1^ The tunable structures and exceptionally high surface areas of MOFs have enabled various applications in gas storage, separations, catalysis, sensing, and drug delivery.^2–7^ A particularly attractive subclass of MOFs is switchable MOFs that can undergo solid–solid phase transformations such as gate opening in response to external stimuli (e.g., guest molecules, pressure, temperature), thereby enhancing gas uptake properties, improving selectivity in chemical separations, increasing catalysis efficiency, and enabling stimuli-responsive drug delivery applications.^8–11^ To enable efficient use of switchable MOFs in industrial applications, it is important to understand mechanical robustness under various operating conditions. Furthermore, size reduction of MOFs toward micro- or nanoscale dimensions is an emerging approach to modify and improve properties of MOFs, such as gas adsorption and framework flexibility, as well as gate-opening transitions.^10,12–14^ However, micro- and nanodimensional MOFs often display properties that can differ from their macroscopic counterparts.^10,14–16^ For example, changes in gas adsorption and gate-opening behavior or even the complete suppression of the gate-opening transition is observed for some switchable MOFs (e.g., DUT-8) as a result of size reduction to submicron dimensions.^10^ While the exact origin of these size-dependent findings is still not well understood, it is frequently attributed to changes in the surface-to-volume ratio and differences in the relative number of point defects (e.g., missing metal nodes and/or organic linkers) as a result of size reduction of switchable MOF crystals at the nanoscale.^10,14,17^ The findings highlight the extent of studies on additional switchable MOFs, as well as methods for characterization ideally on a single-crystal basis, to elucidate effects of size reduction toward the nanoscale on their physicochemical properties, which can then inform design.

One of the questions the present work aims to assess is whether previously observed continuous increase in the framework flexibility of switchable ZIF-7 crystals with size reduction to the nanoscale reflects a general feature of switchable MOFs. Addressing this question requires first establishing whether another chemically and structurally distinct switchable MOF system also exhibits comparable size-dependent framework flexibility and second determining whether the underlying origin of size-dependent response can also be attributed to point defects. Such a comparison can also provide further insights into whether defect accumulation with crystal downsizing is comparatively different between various MOFs.

To evaluate the size-dependent mechanical behavior of switchable MOFs, we selected ZIF-90 as a model system for several reasons. First, we investigated size-dependent mechanical behavior in another switchable MOF ZIF-7.^14^ While both ZIF-7 and ZIF-90 are based on the same Zn^2+^ ion nodes, the organic linkers and crystal structures are different; specifically, benzimidazole versus carboxaldehyde-2-imidazole as the linker and trigonal versus cubic crystal structure for ZIF-7 and ZIF-90, respectively. Second, both frameworks exhibit gate-opening transitions characteristic of switchable MOFs.^18–20^ Finally, ZIF-90 can be synthesized in a facile manner across a wide range of crystal sizes with high chemical and thermal stability, enabling the size-dependent studies presented below. Importantly, the differences in linkers and crystal structures are expected to have different influences on the defect formation and propagation mechanisms during crystal growth, which may, in turn, impact size-dependent mechanical behavior. Collectively, these factors make ZIF-90 a highly suitable model system to probe how differences in the linker type and crystal structure influence the size-dependent mechanical properties of switchable MOFs. We hypothesize that crystal downsizing leads to a continuous increase in the framework flexibility of ZIF-90 through a size-dependent accumulation of missing metal node defects, and that comparison of the power-law exponents between the two systems provides a quantitative measure of the relative rate of defect accumulation as a function of crystal size.

As part of our work, we also introduce a new experimental approach based on single-crystal optical photothermal infrared (OPTIR) spectroscopy measurements that were utilized to further confirm the presence of missing metal node defects in ZIF-90 crystals. OPTIR is an emerging vibrational spectroscopic technique that combines the chemical specificity of infrared absorption with the submicrometer spatial resolution of visible-light microscopy.^21–23^ In OPTIR, a tunable mid-infrared laser excites molecular vibrations, and the resulting photothermal response generated by infrared absorption is detected using a co-aligned visible probe laser.^23–25^ Because the spatial resolution is determined by the visible probe beam rather than the much longer infrared wavelength, OPTIR overcomes the spatial resolution limitations of a conventional IR microscope and enables submicrometer chemical characterization of individual micro- and nanocrystals that are difficult to analyze using conventional methods.^23,24^ Recent nanoscale infrared spectroscopy studies have further demonstrated that localized vibrational spectroscopy can be used to identify missing linker, missing metal, and dangling linker defects in individual MOF crystals through characteristic changes in their vibrational spectra.^23,26,27^ Therefore, single-crystal OPTIR provides a suitable approach for probing localized defect-associated vibrational features in ZIF-90, as these defects may be difficult to distinguish in conventional bulk infrared measurements.^26,27^ Moreover, as OPTIR spectra exhibit a strong correlation with conventional transmission-mode IR spectra, established vibrational mode assignments can be directly applied for the interpretation of defect-associated infrared peaks in single-crystal OPTIR measurements.^23^

In our current study, we show that the reduction in ZIF-90 crystal size from micro- to nanodimensions results in almost a 1 order of magnitude increase in framework flexibility (i.e., reduction of Young’s modulus) as determined by atomic force microscopy (AFM) nanoindentation. A continuous increase in framework flexibility of individual crystals with crystal-size reduction toward the nanoscale is observed, following a power-law dependence. To understand the origin of this size-dependent mechanical behavior, we utilized single-crystal scanning electron microscopy (SEM) and energy-dispersive X-ray spectroscopy (EDX) to analyze elemental composition variations associated with point defect formation. Furthermore, single-crystal OPTIR spectroscopy was established as a new experimental method to examine defect-associated vibrational features in individual ZIF-90 crystals and to relate these changes to the size-dependent response. The origin of the power-law size-dependent mechanical behavior is attributed to an increase in the relative number of point defects (i.e., missing metal nodes) as crystal size decreases. The value of the power-law exponent in the Young’s modulus versus crystal-size data was related to the magnitude of the relative increase in the number of missing metal node defects, indicating that the extent of the framework flexibility change with crystal size can in part be attributed to the rate of defect accumulation with crystal downsizing. These observations suggest that defect-mediated framework softening may be a general feature in switchable MOFs and may depend on framework chemistry and structure. Collectively, our results highlight the critical role of point defects in modulating the framework flexibility of switchable MOFs and in turn influencing their physicochemical properties (e.g., guest-induced gate-opening transitions).

## Experimental Section

### Materials and Synthesis

NaCO_2_H (99%) and Zn­(NO_3_)_2_·6H_2_O (99%) were obtained from Sigma-Aldrich (St. Louis, MO), while methanol (HPLC-grade) and OHC-IM (99%) were obtained from Alfa Aesar (Ward Hill, MA). All compounds were used without further purification. The synthesis of ZIF-90 crystals was performed as described previously.^18^ Briefly, a solution of 20 mmol of NaCO_2_H and 20 mmol of OHC-IM ligand in 50 mL of methanol was prepared and then heated to 50 °C to fully dissolve both solids, followed by letting the ligand solution cool to room temperature (ca. 20 °C). Then, 5 mmol of Zn­(NO_3_)_2_·6H_2_O solution in 50 mL of deionized water was added to the ligand solution. The solution was then stirred for 1 h at room temperature, yielding a cloudy suspension. Next, the suspension was centrifuged at 10000 rpm for 5 min, yielding an off-white precipitate. The resulting precipitate was redispersed twice in 45 mL of methanol, each time followed by centrifuging at the same conditions as above. The resulting powder was then dried in an oven at 85 °C.

### Powder X-ray Diffraction (PXRD) Measurements

PXRD data were obtained at room temperature (ca. 20 °C) on a Bruker D500 X-ray diffractometer using Cu Kα1 radiation (λ = 1.54056 Å); scan type: locked coupled; scan mode: continuous; step size: 0.02°; and scan time: 2 s per step.

### Atomic Force Microscopy (AFM) Single-Crystal Imaging and Nanoindentation Measurements

Powdered ZIF-90 crystals were suspended in methanol (ca. 10 mg of solid in 10 mL of methanol) and drop-cast on a freshly cleaved atomically flat mica surface (V-1 grade, SPI Supplies, Westchester, PA); the solvent was then allowed to evaporate. All AFM studies were performed using a Molecular Force Probe 3D AFM (Asylum Research, Santa Barbara, CA). AFM height images and nanoindentation measurements were performed at room temperature (ca. 20 °C) and ambient pressure using Si_3_N_4_ probes with a hard diamond-like carbon coating (Mikromasch, San Jose, CA) with a nominal spring constant range of 2–5.4 N/m and a tip radius of curvature of <20 nm. Actual spring constants were determined using a built-in thermal noise method.^28^ AFM height images of individual crystals were collected in an AC mode (intermittent contact mode) at a typical scan rate of 1 Hz. The crystal height and crystal size were determined from AFM single-crystal 3D height images. The crystal size (base size) was defined as the geometric mean of the length of the longest crystal axis and the width perpendicular to the longest crystal axis.

AFM nanoindentation experiments were performed by first recording force versus vertical piezo displacement curves (i.e., force curves) at an approximate center of a flat region for an individual substrate-deposited ZIF-90 crystal of interest that was first located via AFM height imaging. Repeated force curves at various positions on the crystal surface (typically 3–8 per crystal) were recorded for each crystal. Measurements and analysis were performed on 34 different individual crystals with base size ranges of 0.4–5 μm and heights of 0.1–2.5 μm. Sizes were determined from single-crystal AFM height images. Images of individual crystals before and after force measurements were performed to ensure there were no apparent changes in the crystal surface because of nanoindentation measurements. The acquisition of force curves and corresponding data analysis was carried out as reported previously.^29–33^ Briefly, the force curves were first converted into force versus indentation distance data, and then the approach to crystal surface data were fit to the Johnson–Kendall–Roberts (JKR) elastic contact model^34^ to determine the Young’s modulus. Young’s modulus values obtained from individual force curves were then averaged to determine the average Young’s modulus for each crystal, with the corresponding standard deviation of the mean representing the variability across multiple force measurements on a particular crystal.

### Scanning Electron Microscopy (SEM) and Energy-Dispersive X-ray Spectroscopy (EDX) Measurements

SEM images were collected using a JEOL JSM-1T700HR Scanning Electron Microscope. Powdered ZIF-90 crystals were directly deposited onto a Si wafer and imaged at 2 keV and 15,000 magnification with a working distance of 6 mm between the sample and electron source. EDX spectra were collected at 20 keV at an approximate center of each crystal. In total, four individual crystals with base sizes ranging from 0.5 to 5 μm were studied. The crystal size (geometric mean) was determined from SEM images of individual crystals.

### Optical Photothermal Infrared (OPTIR) Single-Crystal Measurements

For these measurements, a powdered ZIF-90 sample was suspended in methanol (ca. 10 mg in 10 mL), then drop-cast on an IR-grade CaF_2_ microscope slide (UQG Ltd., England), and methanol was allowed to evaporate prior to OPTIR measurements. Single-crystal measurements were performed using a mIRage microscope (Photothermal Spectroscopy Corp., Santa Barbara, CA, USA). Before individual crystal IR spectrum collection, optical images of individual crystals were recorded using a Cassegrain objective with high magnification (×50). A single-crystal IR spectrum was collected from an approximate center of selected individual crystals using a 40× objective with a 532 nm probe laser. The crystal size (geometric mean) of individual crystals was determined from the optical images using PTIRStudio 5.0.1 software supplied with the instrument.

## Results and Discussion

The formation of structurally pure ZIF-90 crystals was confirmed by PXRD measurements, where good agreement was observed between the calculated and experimental patterns (Figure 1).^18^ AFM imaging of substrate-deposited ZIF-90 sample revealed the presence of individual ZIF-90 crystals displaying prism-like morphologies (Figure 2a) with the base size and height ranges of ∼0.4–5 μm and ∼0.1–2.5 μm, respectively. Similar ZIF-90 morphologies were also observed previously using SEM.^35,36^ Comparatively, ZIF-7 individual crystals similarly displayed prism-like morphologies for microsized crystals.

**Figure 1:**
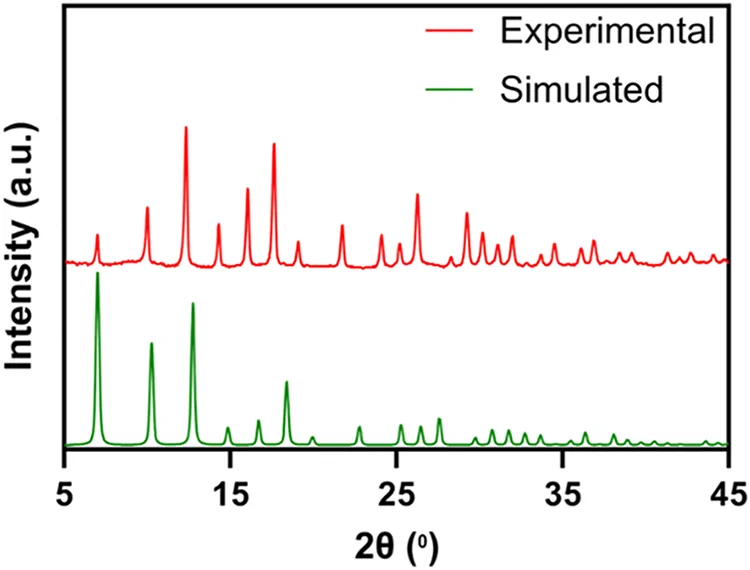
Comparison of ZIF-90 PXRD patterns: simulated pattern from single-crystal X-ray diffraction data^18^ (green) and experimental pattern (red).

**Figure 2:**
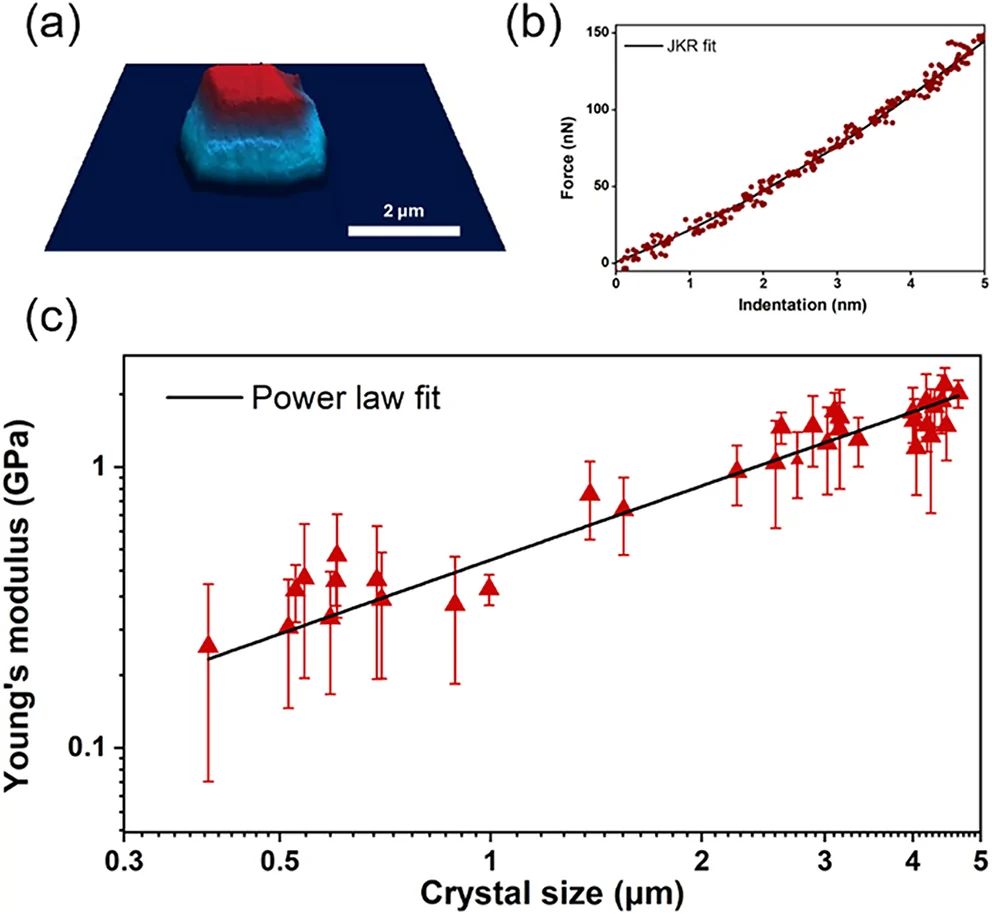
(a) Representative AFM 3D height image of individual ZIF-90 crystals displaying a prism-like morphology with a base size of ∼2 μm and height of ∼1.4 μm. (b) Representative force–indentation plot (only the approach to the crystal surface data is shown) collected at an approximate center of the crystal shown in (a), where circles represent the data and the solid line is the fit using the JKR model, yielding a Young’s modulus value of 1.4 GPa ± 0.1 GPa. (c) Average Young’s moduli (triangles) and one standard deviation (error bars) as a function of crystal size (log–log scale) for individual ZIF-90 crystals, where the solid line represents a power-law fit.

Figure 2b shows a representative force–indentation plot (circles represent the approach to the crystal surface data) obtained at an approximate center of the crystal shown in Figure 2a. The force–indentation data were fit to the JKR model,^34^ yielding the Young’s modulus and one standard deviation of 1.4 GPa ± 0.1 GPa for the single force measurement. For individual crystal shown in Figure 2a, three repeated force–indentation measurements were performed at different crystal surface positions, followed by the JKR fit for each force plot, yielding the average Young’s modulus and one standard deviation of the mean of 1.4 ± 0.2 GPa. These measurements were similarly extended to a total of 34 individual ZIF-90 crystals with base sizes ranging from ∼0.4 to ∼5 μm and the corresponding heights from ∼2.5 μm to ∼0.1 μm. Figure 2c shows the average Young’s modulus of individual crystals as a function of the corresponding crystal size (log–log axis), where each error bar represents one standard deviation of the mean. As can be seen from Figure 2c, the Young’s moduli of ZIF-90 crystals exhibit a clear size-dependent trend, where crystals become softer (lower Young’s modulus) as the crystal size decreases. Specifically, reduction in crystal size from ∼5 μm to ∼0.4 μm results in almost 1 order of magnitude decrease in Young’s modulus (from 1.8 ± 0.2 GPa to 0.23 ± 0.15 GPa), implying a remarkable increase in framework flexibility at nanoscale. Note that the average Young’s modulus obtained for the largest ZIF-90 crystals examined in this study (1.8 ± 0.2 GPa, base size ∼5 μm) is comparable to the value of 2.05 GPa calculated for a defect-free, bulk ZIF-90 framework using density functional theory,^37^ indicating that the largest crystals studied here approach the mechanical behavior expected for a largely defect-free framework. Furthermore, the plot in Figure 2c clearly shows a linear trend, implying a power-law dependence between the average Young’s modulus and crystal size. Thus, the data were fit to a power-law function *E* = α*D* ^β^, where *E* (GPa) is the average Young’s modulus, *D* (μm) is the crystal base size, and α (proportionality constant) and β (power-law exponent) are free fit parameters. Fit yields α = 0.50 ± 0.02 GPa and β = 0.88 ± 0.05, with *R* ^2^ = 0.92.

For comparison, studies on ZIF-7 have also shown a similar power-law dependence between Young’s modulus and crystal size, indicating that such scaling behavior may extend across different switchable MOF systems.^14^ In the case of ZIF-7, a power-law fit yielded power-law exponent β = 0.61 ± 0.05, which is clearly lower than that observed for ZIF-90 above. The fact that both systems display power-law dependence is remarkable owing to distinct differences in organic linkers (benzimidazole versus carboxaldehyde-2-imidazole) and crystal structures (trigonal versus cubic) for these two MOFs. Thus, this observation indicates that the observed power-law mechanical properties dependence may represent a general trend that potentially extends to other types of switchable MOFs. We next discuss the origin of the observed size-dependent reduction in the Young’s modulus and rationalize observed differences in the power-law exponent between these two systems by employing complementary single-crystal SEM EDX measurements.

Figure 3a shows a representative SEM image of individual prism-like ZIF-90 crystals, displaying morphologies consistent with AFM results presented above. SEM EDX spectra (Figure 3b) were collected on four ZIF-90 crystals with base sizes ranging from ∼0.5 to 5 μm. As shown in Figure 3b, both the carbon K edge (0.27 keV) and zinc L edge (1.02 keV) signals decrease in intensity with decreasing crystal size, as expected. Because the zinc L edge intensity corresponds to the number of metal nodes in the framework, and the carbon K edge intensity represents the number of organic linkers, the Zn/C ratio provides a relative assessment of metal-to-ligand composition. Figure 3c shows the carbon K edge EDX spectra of individual crystals normalized to the corresponding carbon K edge signal, where a clear reduction in the carbon-normalized zinc signal is observed for smaller crystals, implying a lower Zn/C ratio for smaller ZIF-90 crystals (Figure 3d). This trend closely parallels that observed previously for ZIF-7, where it similarly showed a reduction in the carbon-normalized zinc signal for smaller crystals, which was rationalized via an increased concentration of missing metal node defects as crystals downsize to nanoscale dimensions.^14^ Moreover, the linear fitting of the Zn/C ratio versus crystal-size data yielded a slope of 0.26 ± 0.03 μm^–1^ with *R* ^2^ of 0.97 for ZIF-90 (Figure 3d) compared to the fitted slope of 0.05 μm^–1^ ± 0.01 μm^–1^ (*R* ^2^ of 0.95) for similarly obtained Zn/C ratio versus crystal-size data for the ZIF-7 system (Figure 4), confirming that the compositional change with crystal downsizing is significantly steeper in ZIF-90.^14^ We note that the observed Zn/C ratio size-dependent reduction trend with crystal size decrease could, in principle, result from either a reduction in the Zn signal (more missing metal node defects) or an increase in carbon (few missing linker defects). However, the latter explanation is not consistent with our observed reduction of the Young’s modulus with crystal downsizing, which can be attributed to an expected increase in the relative number of point defects. Thus, the observed power-law reduction in the Young’s modulus with crystal size decrease is attributed to an increase in the relative number of missing metal nodes for smaller crystals at the nanoscale.

**Figure 3:**
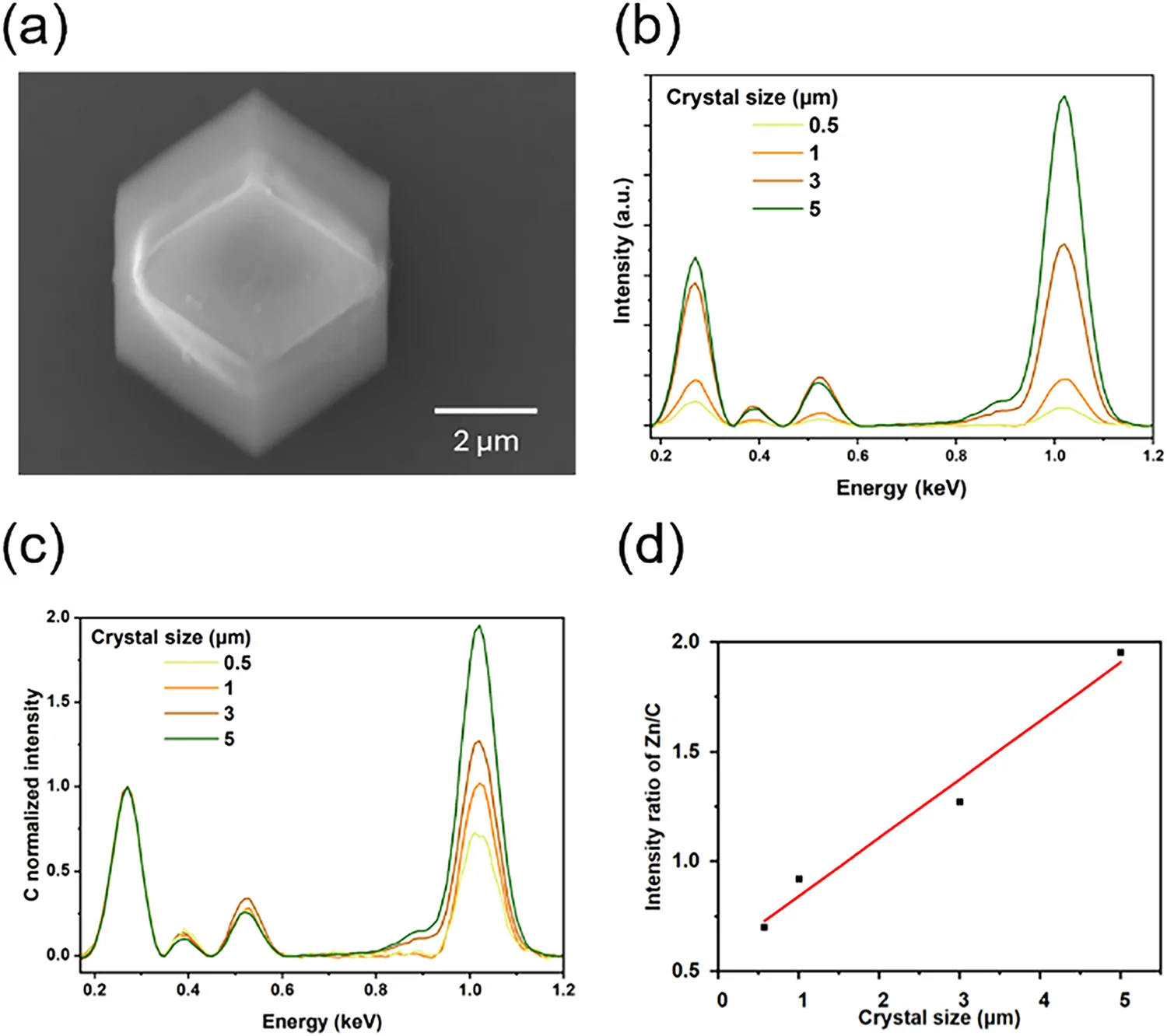
(a) Representative SEM of individual ZIF-90 crystals displaying prism-like morphologies with a base size of ∼5 μm. (b) Absolute and (c) C-normalized EDX spectra of several selected individual ZIF-90 crystals of various base sizes. (d) EDX peak intensity ratio of Zn/C as a function of ZIF-90 crystal size; symbols represent individual crystal data, and the solid red line corresponds to a linear fit.

**Figure 4:**
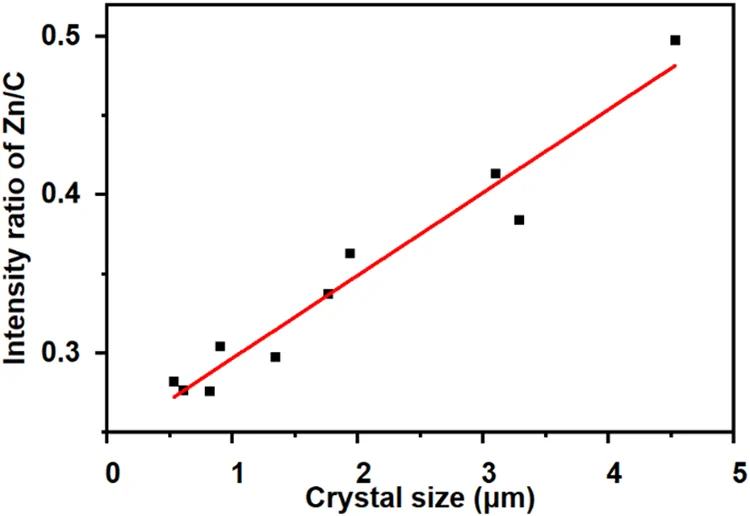
EDX peak intensity ratio of Zn/C as a function of ZIF-7 crystal size; symbols represent individual crystal data, and the solid red line corresponds to a linear fit. Reproduced from ref 14 with permission from the Royal Society of Chemistry, Copyright 2024.

To further corroborate the presence of missing metal node defects inferred from the size-dependent EDX analysis presented above, OPTIR spectroscopy measurements were performed for the first time to probe the local coordination environment of individual ZIF-90 crystals. Figure 5 shows the optical image and the corresponding OPTIR spectrum of representative ZIF-90 crystals. The crystal selected for spectral acquisition is indicated by the red arrow. The analyzed crystal exhibits a base size of 3 μm, as determined from the optical image analysis. The corresponding OPTIR single-crystal infrared spectrum shows excellent agreement with previously reported bulk IR spectra of ZIF-90, confirming the preservation of the framework structure at the single-crystal level.^38,39^ Notably, the characteristic absorption band at **∼**1668 cm^–1^, assigned to the C=O stretching vibration of the aldehyde in the imidazole-2-carboxaldehyde ligand, is clearly observed. In addition, bands at 1456, 1416, 1362, and 1168 cm^–1^ are consistent with vibrational modes of the imidazole moiety, further supporting the structural and chemical integrity of the framework.^38–40^

**Figure 5:**
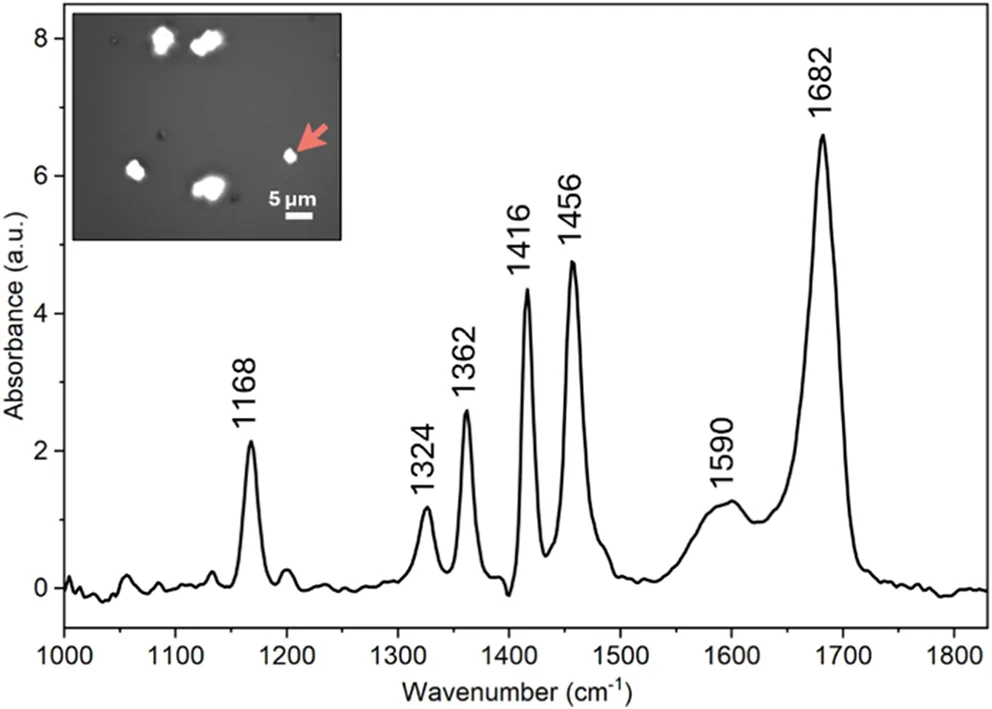
OPTIR characterization of ZIF-90 single crystals. Optical microscopy image (inset) of representative crystals deposited on a CaF_2_ substrate, where the red arrow indicates the crystal over which the single-crystal OPTIR spectrum shown in the figure was collected, displaying characteristic vibrational features of ZIF-90.

Formation of the ZIF framework involves the deprotonation of the imidazole nitrogen to enable coordination with Zn^2+^ centers, and therefore, a fully coordinated structure is not expected to exhibit significant N–H vibrational features.^41^ Deviations from this ideal coordination environment can therefore be probed through the appearance of N–H-related vibrational features.^4–6^ This behavior has been demonstrated in nanoscale IR studies of ZIF systems. In particular, Möslein et al. showed that vibrational modes associated with C–N–H groups of uncoordinated ligands are prominent in defective ZIF-8 crystals and decrease as crystallization proceeds and stable Zn–N coordination is established, indicating that these features arise from dangling ligands associated with missing metal nodes.^26^ In parallel, detailed vibrational and computational analysis by Ahmad et al. demonstrated that missing Zn defects lead to protonated ligand sites (−C–N–H), giving rise to distinct N–H bending modes, with bands in the ∼1580–1590 cm^–1^ region attributed to such defect-associated ligands and absent in defect-free structures.^27^ In this context, the additional band observed at ∼1590 cm^–1^ in the OPTIR single-crystal spectrum of ZIF-90 is assigned to N–H bending associated with protonated, undercoordinated ligand sites. The band suggests local disruption of metal–ligand coordination, consistent with the presence of missing Zn defects. Thus, the 1590 cm^–1^ band provides strong evidence for missing metal node defect sites in ZIF-90 single crystals, consistent with the EDX analysis presented above.

We note that while both ZIF-7 and ZIF-90 MOF systems exhibit a power-law decrease in Young’s modulus with crystal downsizing that we attribute to an increase in the relative number of missing metal nodes, the power-law exponents are clearly different (0.61 for ZIF-7 versus 0.88 for ZIF-90). This indicates that the extent of Young’s modulus reduction with crystal size decrease is significantly greater for ZIF-90. In addition to the difference in the Young’s modulus power-law exponents, the Zn/C ratio trends also show markedly different fitted slopes, with 0.26 μm^–1^ for ZIF-90 and 0.05 μm^–1^ for ZIF-7. Together, these results may indicate that the extent of defect accumulation with crystal downsizing is more pronounced in ZIF-90 than in ZIF-7, consistent with the greater mechanical softening observed for ZIF-90. Collectively, based on the presented ZIF-90 and previously reported ZIF-7 findings, these results confirm the importance of point defects on the properties of MOFs and establish that the relative concentration of missing metal node defects increases with crystal-size reduction, impacting the framework flexibility and suggesting that such behavior is possibly a general phenomenon in switchable MOFs and likely represents a key factor that governs their size-dependent properties. These findings further suggest that the power-law scaling of mechanical properties in MOFs is closely linked to defect evolution, and such information may provide a framework to compare size-dependent defect accumulation efficiency across different switchable MOF systems.

## Conclusions

In summary, AFM single-crystal nanoindentation measurements revealed that ZIF-90 crystals show a continuous power-law decrease in crystals’ Young’s modulus (i.e., increasing framework flexibility) with crystal-size reduction to the nanoscale. Combined single-crystal AFM, SEM/EDX, and OPTIR measurements indicate that the origin of the observed increase in the framework flexibility of ZIF-90 crystals with crystal-size reduction is related to the relative increase in missing metal node defects for smaller crystals. Furthermore, the magnitude of the power-law exponent, which indicates the extent of the framework softening with size reduction, can be linked to the relative rate of defect accumulation as a function of crystal size. Overall, these results highlight how the generation and relative concentration of point defects vary with crystal structure and size, in turn governing the degree of size-dependent framework flexibility in switchable ZIF systems. Extending these studies to other switchable MOFs with different metals, organic linkers, and topologies will be essential for developing a better understanding of the extent of size-dependent variability of point defects, ultimately opening a pathway toward a rational design and control over the framework flexibility in MOFs through crystal-size engineering.
